# Supplementary material for: Violation of detailed balance in non-equilibrium magnons observed by inelastic neutron scattering
Source: Nat Commun. 2026 Apr 16;17:3535. doi: 10.1038/s41467-026-71068-w (PMC13087290; doi:10.1038/s41467-026-71068-w)
Supplement: Supplementary file 1 — Supplementary Information [file 41467_2026_71068_MOESM1_ESM.pdf]

Supplementary Materials for
Violation of detailed balance in non-equilibrium magnons observed by inelastic neutron scattering

C. Hua,[1] B. Winn,[2] C. Sarkis,[2] Gabriele Sala,[3] T. Egami,[1,4,5,6] and D.A. Tennant[4,5,6]

[1]*Materials Science and Technology Division, Oak Ridge National Laboratory, Oak Ridge TN, 37831, USA*

[2]*Neutron Scattering Division, Oak Ridge National Laboratory, Oak Ridge TN, 37831, USA*

[3]*Japan Proton Accelerator Research Complex, Tokai, Ibaraki 319-1106, Japan*

[4]*Department of Physics and Astronomy, University of Tennessee, Knoxville, TN 37996, USA*

[5]*Department of Materials Science and Engineering, University of Tennessee, Knoxville TN, 37996, USA*

[6]*Shull Wollan Center, Oak Ridge National Laboratory, Oak Ridge TN, 37831, USA*

(Dated: March 10, 2026)

CONTENTS

I. INS Data and uncertainty analysis 2

A. Measurements at equilibrium 2

B. Measurements with laser excitation 3

C. Overheating examples 8

II. Classical theory on non-equilibrium steady states 11

A. Two-in two-out magnon-magnon scattering 11

B. Distribution function in a closed magnon system 12

C. Nonequilibrium steady states under an external periodic drive 13

III. Quantum theory on broken detailed balance 15

References 19

I. INS DATA AND UNCERTAINTY ANALYSIS

A. Measurements at equilibrium

Magnetic neutron scattering measures the Fourier transform pair correlation functions[1]:

$$S^{\alpha\beta}(\mathbf{Q}, E) = \frac{1}{2\pi N} \sum_{l,l'} e^{i\mathbf{Q}\cdot(\mathbf{R}_l - \mathbf{R}_{l'})} \int_{-\infty}^{\infty} e^{-iEt/\hbar} \langle \hat{S}_l^{\alpha}(0) \hat{S}_{l'}^{\beta}(t) \rangle dt, \tag{1}$$

where $\hat{S}_l^{\alpha}$ ($\alpha = x, y, z$) is the spin operator of the lth ion at site $\mathbf{R}_l$. $\langle \hat{S}_l^{\alpha}(0) \hat{S}_{l'}^{\beta}(t) \rangle$ is the thermal average of the time-dependent spin operators and gives the probability that, if the magnetic moment of the lth ion at site $\mathbf{R}_l$ has some specified value at time zero, then the moment of the l'th ion at site $\mathbf{R}_l'$ has some other specified value at time t. At thermodynamic equilibrium, time reversibility places a symmetry constraint on two-time correlation functions given as

$$\langle \hat{S}_l^{\alpha}(0) \hat{S}_{l'}^{\beta}(t) \rangle = \langle \hat{S}_{l'}^{\beta}(0) \hat{S}_l^{\alpha}(i\hbar/(k_B T) - t) \rangle, \tag{2}$$

where k_B is the Boltzmann constant and T is the temperature of the system. Often, Eq. (2) is referred to the principle of detailed balance, which requires the dynamic structure factor following $S^{\alpha\beta}(\mathbf{Q}, -E) = exp(-E/k_B T) S^{\alpha\beta}(\mathbf{Q}, E)$[2]. In a magnetic neutron scattering experiment, $S^{\alpha\beta}(\mathbf{Q}, E < 0)$ corresponds to an annihilation process where a neutron gains energy and annihilates a magnon. The magnitude of $S^{\alpha\beta}(\mathbf{Q}, E < 0)$ is proportional to the Bose-Einstein distribution, $n^{BE}(T)$, at equilibrium. $S^{\alpha\beta}(\mathbf{Q}, E > 0)$ is the creation process where a neutron loses energy and creates a magnon and its magnitude is proportional to $n^{BE}(T) + 1$.

To confirm the principle of detailed balance at equilibrium, we first performed the equilibrium INS measurements at various temperatures: 3.6 K, 6 K, 9 K, 14 K, and 19 K as shown in Fig. 2(a) of main text and Figs. S1(a)-(d). At equilibrium, the total dynamic structure factor of Rb_2MnF_4 can be predicted with high accuracy. We calculated the intensity of the total dynamic structure factor of Rb_2MnF_4 at thermodynamic equilibrium given as

$$\begin{aligned} I(\mathbf{Q}, E) = &\int d\mathbf{Q}' dE' F(\mathbf{Q}')^2 \mathcal{S}(\mathbf{Q}', E') R(\mathbf{Q} - \mathbf{Q}', E - E') \\ &\times \sum_{\mathbf{Q}'} \left\{ \delta(E' - E_{Q'})[n^{BE}(E_{Q'}, T) + 1] + \delta(E' + E_{Q'}) n^{BE}(E_{Q'}, T) \right\} \end{aligned} \tag{3}$$

where $F(\mathbf{Q})$ is the Mn^{2+} magnetic form factor and $\mathcal{S}(\mathbf{Q}, E)$ is the structure factor at $T = 0$ K. The exact expression can be found in Ref. [3]. HYSPEC instrumental resolution function, $R(\mathbf{Q}, E)$ in

Eq. (3), has been modeled employing the software MCViNE[4, 5]. The dynamic structure factor, $S(\mathbf{Q}, E)$, as appeared in the main text at a nonzero temperature is written as

$$S(\mathbf{Q}, E) = \mathcal{S}(\mathbf{Q}, E)\left\{\delta(E - E_Q)[n^{BE}(E_Q, T) + 1] + \delta(E + E_Q)n^{BE}(E_Q, T)\right\}. \tag{4}$$

The modeled $I(Q, E)$ at various temperatures are shown in Figs. S1(e)-(h). To demonstrate the accuracy of the model, Fig. S2 compares energy-dependent cuts between the measured and modeled data at 3.6 K for a series of Q values (Q is along H in $[HHL]$). To show detailed balance between the annihilation and creation intensity is strictly followed at equilibrium (Fig. 2(b) in the main text), we obtained the integrated intensity for magnon annihilation and creation processes separately at various temperatures from both measured and modeled $I(Q, E)$. As demonstrated in Fig. S3, the intensity associated with magnon annihilation process is obtained by integrating the area under the negative side of E-axis ($E \in [-2, -0.3]$ meV) while the creation intensity is obtained by integrating the area under the positive side of E-axis ($E \in [0.3, 2]$ meV). Due to the low incident neutron energy, the cutoff energy transfer is set to be 2 meV. Above 2 meV, the INS data are incomplete in the measured reciprocal space. At equilibrium, the integrated annihilation and creation intensity at different temperatures form a linear relationship with a slope of close to one as shown in Fig. 2(b). The slight deviation of the slope from unity at elevated temperatures may originate from enhanced background noise levels that become more pronounced as the temperature increases.

B. Measurements with laser excitation

Once the baseline for equilibrium is set, we are ready to study the effects of laser excitation. We adapt the notation in Fig. 1(c) and define the number of laser pulses per excitation event as N, number of neutron pulses between two excitation events as j and time between two laser excitation events as P. The laser repetition frequency is fixed at 2000 Hz. Therefore, the maximum number of laser pulses we can fit between two neutron pulses is $N = 33$. In the main text of the current work, we show the laser excited INS data for $N = 30$, $j = 4$ and $P = 51.8$ ms. As shown in Fig. 2(d) of the main text, the intensity on the magnon creation side is well described by the equilibrium data at 3.6 K while an excessive intensity around the zone center was observed only on the magnon annihilation side. Here, Fig. S4 shows that this increase in intensity on the magnon annihilation side is non-thermal and statistically meaningful.

Figures S4(a) & (b) compare the measured intensity at equilibrium and with laser excitation

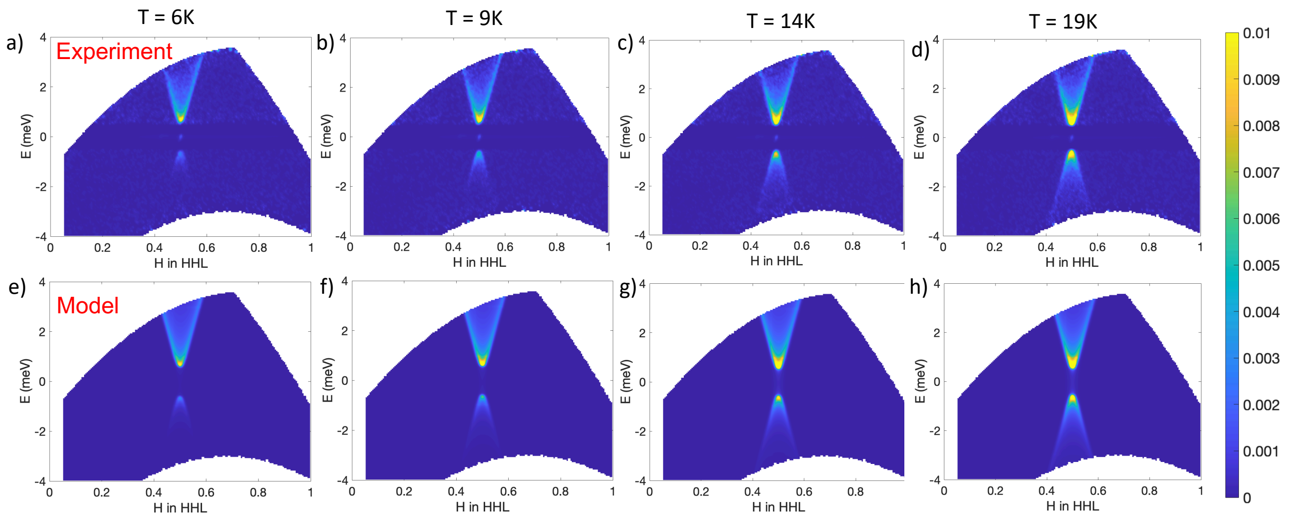

FIG. S1. (a)-(d) Measured (top row) and (e)-(h) modeled (bottom row) $I(Q, E)$ of Rb_2MnF_4 at equilibrium. x-axis is along H in $[HHL]$ and L is integrated between $[2.1, 2.9]$ in reciprocal lattice units (r.l.u.). Ion anisotropy leads to a gap about 0.6 meV. The elastic line ($E \in [-0.3, 0.3]$ meV) is masked off for a clear comparison between the measurement and model. All color maps are on the same scale.

to the modeled intensity at both 3.6 K and 4.6 K. While the modeled data at 3.6 K reproduce the creation-side intensity, the 4.6 K modeled data best reproduce the annihilation-side intensity of the laser-excited spectrum. Here we treat the data as a conventional INS measurement and do not distinguish the difference among different neutron frames. We call such data set as time-integrated. Later we will show the INS data for each neutron frame. The annihilation and creation intensities with laser excitation have to be described by two different temperatures. Figures S4(c) & (d) give the residual intensity when the measured intensity is subtracted by the modeled intensity at both 3.6 K and 4.6 K. Figure S4 (c) show that at an equilibrium temperature of 3.6 K, the residual intensity with respect to the modeled intensity at 3.6 K is around zero on both creation and annihilation sides within the experimental uncertainty. The residual intensity with respect to the modeled intensity at 4.6 K shows negative peaks on both sides, indicating more magnon states will be populated according to Bose-Einstein distribution and can be created and annihilated by neutrons. However, with laser excitation as shown in Fig. S4(d), the residual intensity is around zero only on the annihilation side with respect to the modeled intensity at 4.6 K while the negative peak on the creation side is still present. On the other hand, the residual intensity with respect to the modeled intensity at 3.6 K shows an opposite feature. This observation excludes the excess intensity on the annihilation side with laser excitation is simply due to thermal effects. The apparent over-subtraction

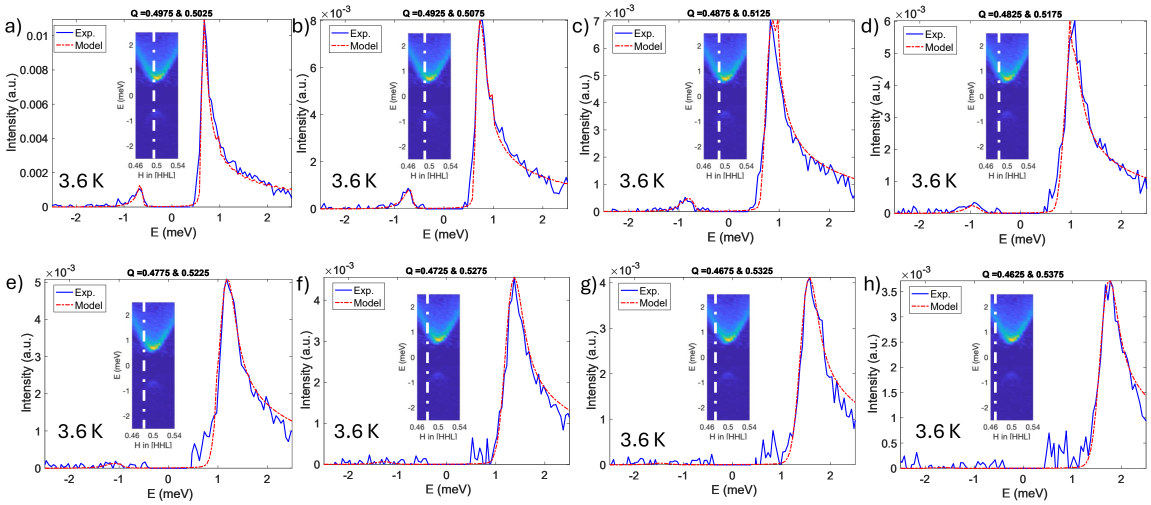

FIG. S2. Energy-dependent cuts between the measured (equilibrium; solid lines) and modeled (dashed lines) data at 3.6 K for a series of Q values. Q is along H in $[HHL]$ and L is integrated between $[2.1, 2.9]$ in reciprocal lattice units (r.l.u.). From (a) to (h), Q incrementally moves away from the magnetic zone center in steps of 0.005 r.l.u.

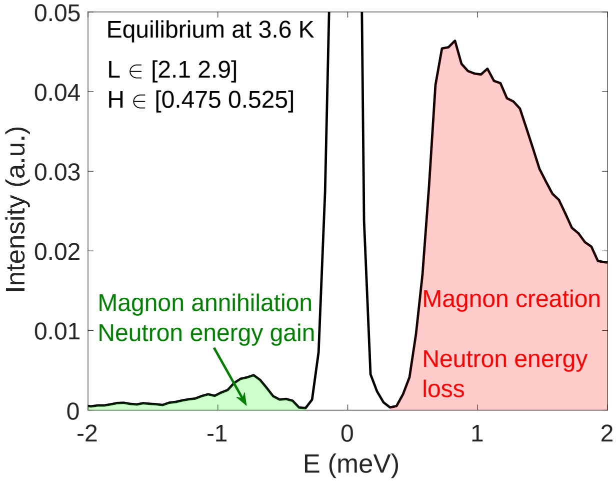

FIG. S3. Equilibrium INS intensity about the zone center as a function of energy transfer, E (meV) at 3.6 K. L is integrated between $[2.1, 2.9]$ (r.l.u.) and H is integrated between $[0.475, 0.525]$ (r.l.u.).

near $E = -0.6$ meV and under-subtraction near $E = -0.8$ meV and a slight over-subtraction over $E > 1$ meV (dashed blue line in Fig. S5(d)), may suggest that the shape of the magnon spectrum is modified under laser excitation. However, these features remain within statistical uncertainty, making it difficult to draw a definitive conclusion.

To investigate any time-dependent information in the laser excited INS data, the data acquired

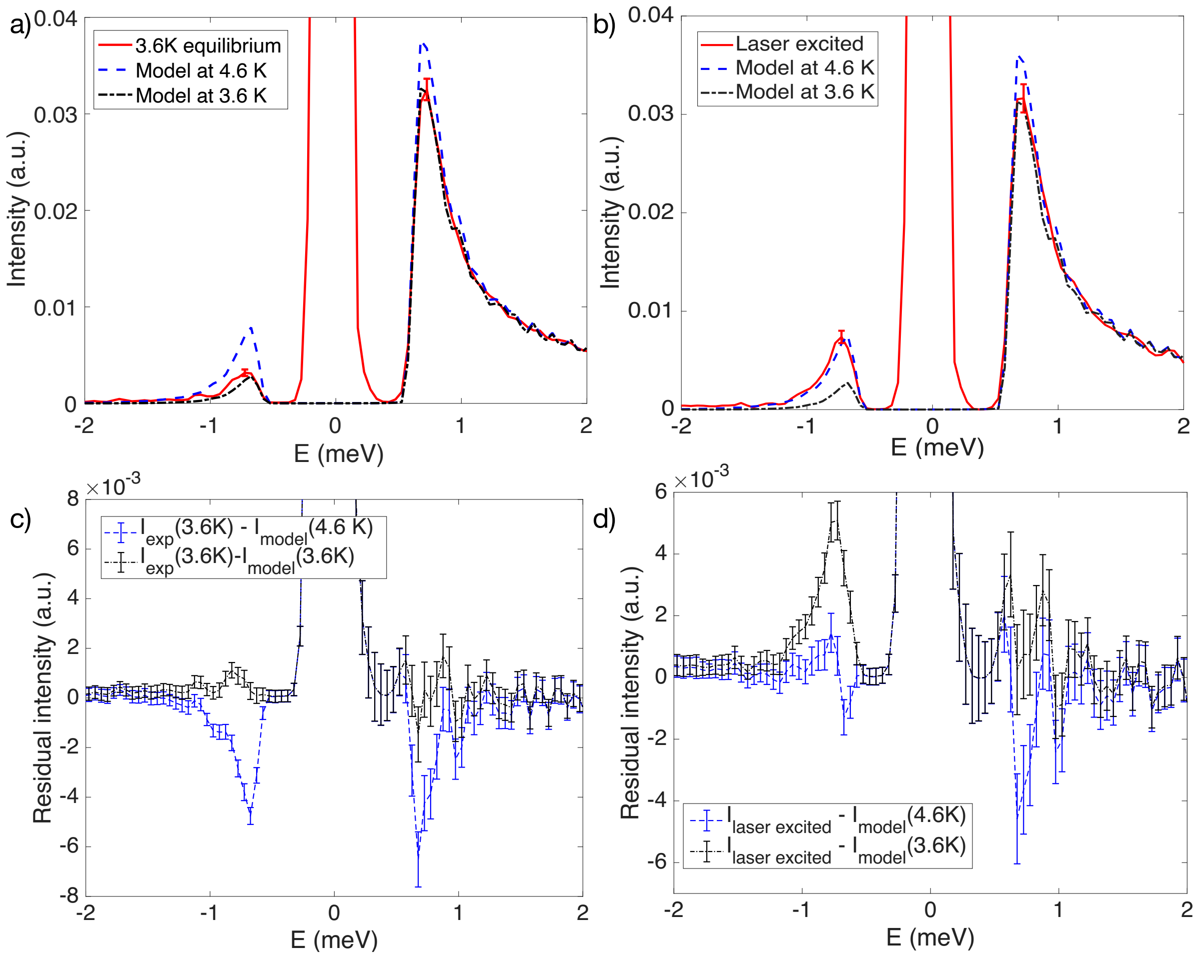

FIG. S4. The measured intensities (solid red lines) (a) at an equilibrium temperature of 3.6 K and (b) with laser excitation are compared with the modeled intensity at both 3.6 K (dot-dashed black lines) and 4.6 K (dashed blue lines). L is integrated between $[2.1, 2.9]$ (r.l.u) and H is integrated between $[4.9, 5.1]$. The residual intensity as a function of energy transfer E is calculated by subtracting the measured intensity (c) at the equilibrium temperature and (d) with laser excitation with the modeled intensity at both 3.6 K (dot-dashed black lines) and 4.6 K (dashed blue lines). All error bars denote $\pm 1\sigma$ uncertainties derived from Poisson neutron counting statistics ($\sigma = \sqrt{N}$).

for each equivalent frame is reduced separately. The details of firmware and software support for data reduction can be found in Ref. [5]. Figure S5 shows the integrated annihilation and creation intensity under laser excitation over four neutron frames between two consecutive laser excitation events. No decay is observed across the four neutron frames, indicating steady-state behavior.

To get a comprehensive understanding of the dynamical behaviors of non-equilibrium magnon states, we conducted a series of laser excited INS measurements with different neutron pulse

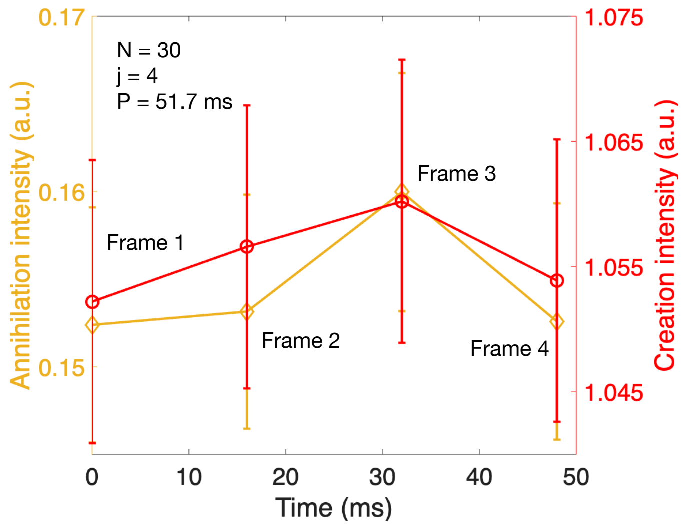

FIG. S5. Magnon annihilation (diamonds; left y axis) and creation (circles; right y axis) intensities measured over four neutron frames between two consecutive laser excitation events. No decay is observed across the four neutron frames, indicating steady-state behavior. The number of laser pulses per pumping event is set to N=30, and the number of neutron pulses incident on the sample between consecutive laser pumping events is $j = 4$, resulting in $P \sim 51.7$ ms. The data are integrated over $H \in [0.475, 0.525]$ and $L \in [2.1, 2.9]$ r.l.u; Energy integration ranges are $E \in [-2, -0.3]$ meV for annihilation and $E \in [0.3, 2]$ meV for creation. All error bars denote $\pm 1\sigma$ uncertainties derived from Poisson neutron counting statistics ($\sigma = \sqrt{N}$).

number between two laser excitation events: $N = 10$ is fixed and P =61.8, 78.5, 95.2, and 262.2 ms. This set of studies was conducted a year later at HYSPEC. Due to slight different experimental conditions (cryostat Helium flow setting and sample surface degradation), we lowered the number of laser pulses to be 10 instead of 30 to avoid heating issue. We estimated here that the total energy deposited onto the sample per laser excitation event is similar between the two experimental setting such that comparison among measurements remains valid. Figures S6 (a)-(c) compare the measured annihilation intensity with laser excitation (time-integrated) and at an equilibrium temperature of 3.6 K and Figs. S6 (d)-(e) compare the corresponding creation intensity. For P = 61.8, 78.5, and 95.2 ms, excess intensity on the annihilation side is observed. The excess intensity decreases as P increases until it reaches the equilibrium value at 3.6 K when P = 262.2 ms. On the creation side, the intensity with laser excitation is well described by the equilibrium intensity at 3.6 K. Again, no statistically meaningful decay was observed between two laser excitation events for all the cases presented here. For example, Fig. S7(a) compares the INS intensities of the first and last neutron

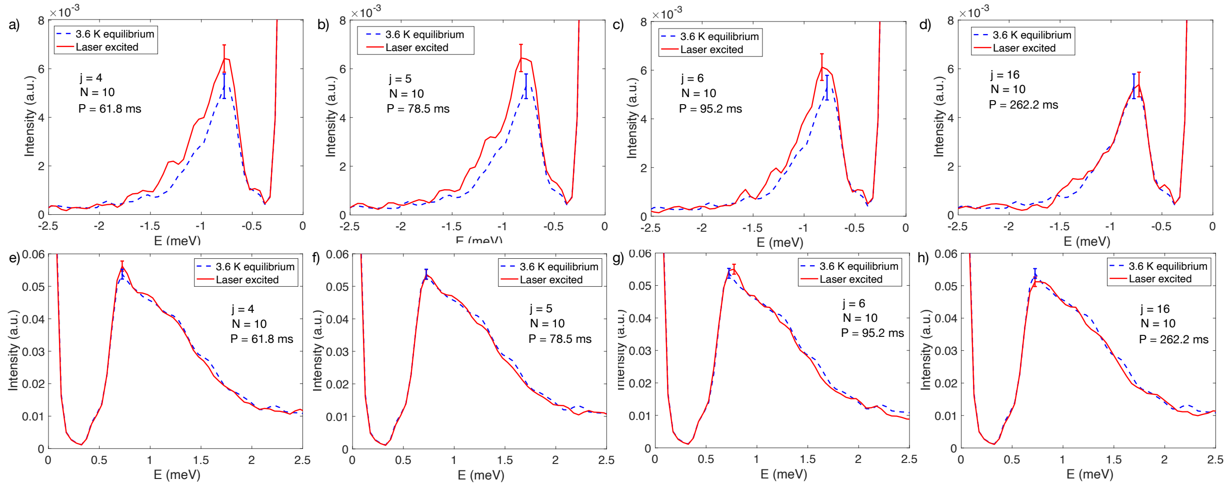

FIG. S6. Comparison between the time-integrated intensity with laser excitation (solid red lines) and equilibrium measurement at 3.6 K (dashed blue line): (a)-(d) annihilation intensity as a function of energy transfer ($E < 0$); (e)-(h) creation intensity as a function of energy transfer ($E > 0$). L is integrated between $[2.1, 2.9]$ and H is integrated between $[0.475, 0.525]$ (r.l.u.). Here, N is fixed at 10 and j (or P) varies by column. Excess intensities on the annihilation side are observed when $P = 61.8$, 78.5, and 95.2 ms but returned to 3.6 K equilibrium value when $P = 262.2$ ms. Representative error bars at ± 0.6 meV denote $\pm 1\sigma$ uncertainties derived from Poisson neutron counting statistics ($\sigma = \sqrt{N}$).

frames when laser excitation scheme is set to be $N = 10$ and $P = 262.2$ ms. The intensity remains unchanged within 262.2 ms. Figure S7(b) shows the integrated annihilation and creation intensities over sixteen neutron frames between two consecutive laser excitation events. No time-dependent behavior is observed within 262.2 ms. Additionally, Fig. S8 shows that the magnetic Bragg peaks remain unchanged, verifying that the long-range magnetic structure is not perturbed by the laser excitation.

C. Overheating examples

An estimated temperature increase of 5 K, mentioned in Sec. **??**, represents a worst-case operating condition, corresponding to continuous laser excitation at the maximum power and a repetition rate of 2000 Hz. In the laser-excited INS measurements discussed here, the laser power and excitation period were deliberately optimized to avoid such conditions. As a result, no evidence of excessive heating is observed at the base temperature. In particular, the intensity on the magnon

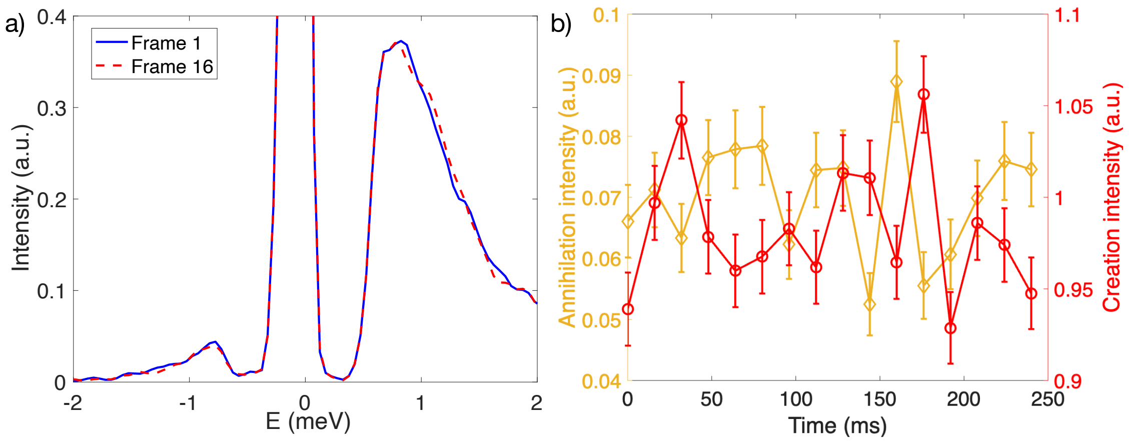

FIG. S7. (a) Comparison between the intensities with laser excitation at two delay times, t_{delay} = 0 ms (solid blue line) and 262.2 ms (red dashed line). Here, the delay time is defined as the difference in arrival time at the sample position between the last laser pulse in a laser excitation sequence and a particular neutron frame in that period. L is integrated between [2.1, 2.9] (r.l.u.) and H is integrated between [0.475,0.525] (r.l.u.). (b) Magnon annihilation (diamonds; left y axis) and creation (circles; right y axis) intensities measured over sixteen neutron frames between two consecutive laser excitation events. The data are integrated over $H \in [0.475, 0.525]$ and $L \in [2.1, 2.9]$ (r.l.u); Energy integration ranges are $E \in$ [-2,-0.3] meV for annihilation and $E \in$ [0.3, 2] meV for creation. Error bars denote $\pm 1\sigma$ uncertainties derived from Poisson neutron counting statistics ($\sigma = \sqrt{N}$).

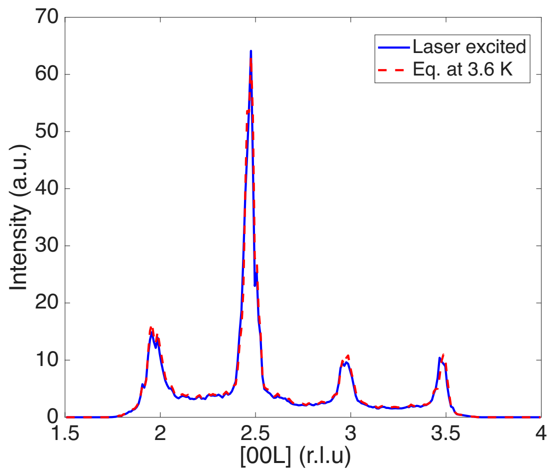

FIG. S8. The measured intensities at $E = 0$ meV at an equilibrium temperature of 3.6 K (red dashed line) and with laser excitation (solid blue line). H is integrated between [0.4, 0.6] (r.l.u.).

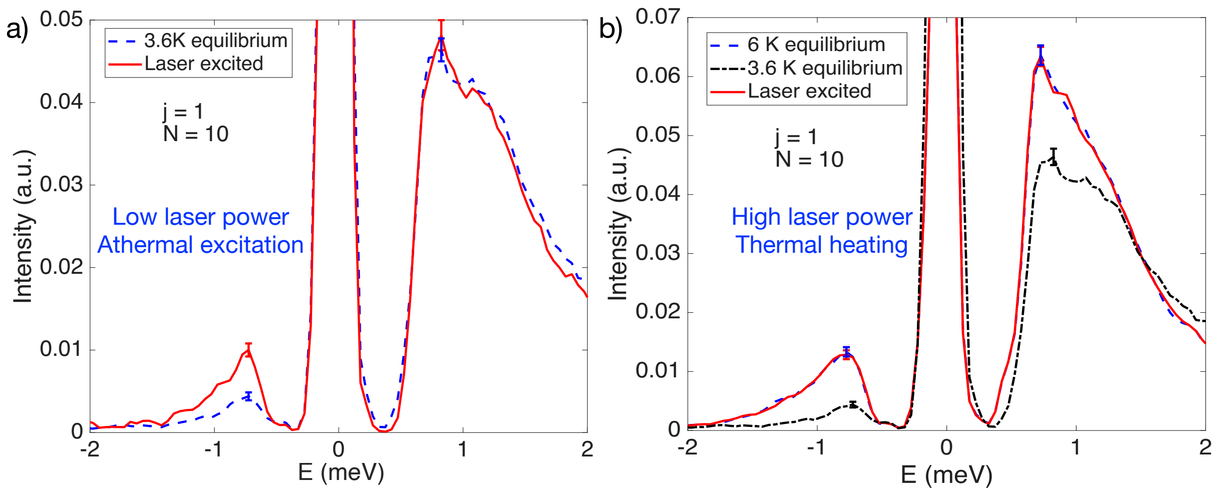

FIG. S9. Laser-excited magnon spectra (solid red lines) measured under (a) low- and (b) high-power laser excitation, with all other excitation parameters kept identical, compared to the equilibrium magnon spectra (dashed lines) measured at 3.6 K and 6 K. L is integrated between [2.1, 2.9] (r.l.u.) and H is integrated between [0.475,0.525] (r.l.u.). Representative error bars at ± 0.6 meV denote $\pm 1\sigma$ uncertainties derived from Poisson neutron counting statistics ($\sigma = \sqrt{N}$).

creation side is unchanged relative to the 3.6 K equilibrium INS data, indicating negligible sample heating. The incident laser power was controlled by rotating a half-wave plate positioned upstream of a polarizing beam splitter.

Sample overheating is expected to manifest clearly in the magnon INS spectra. To demonstrate this effect, an additional figure is provided in the Supplementary Information (Fig. S9), which compares laser-excited spectra measured under low- and high-power laser excitation while keeping all other excitation parameters identical. Under low laser power, the sample remains at the base temperature of 3.6 K, as confirmed by the unchanged creation-side intensity that matches the 3.6 K equilibrium INS spectrum. At the same time, an excess intensity appears on the annihilation side, consistent with the formation of a non-equilibrium magnon population induced by laser pumping.

By contrast, under high laser power, the intensities on both the creation and annihilation sides increase and follow Bose–Einstein statistics. In this regime, the spectra are well described by the 6 K equilibrium INS data, providing a clear and unambiguous signature of sample overheating.

II. CLASSICAL THEORY ON NON-EQUILIBRIUM STEADY STATES

To understand the observed non-equilibrium features in the measurements, we first use Boltzmann transport equation, a classical transport theory, to investigate the dynamical behaviors of nonequilibrium magnon population in Rb_2MnF_4. Out-of-equilibrium magnon transport in Heisenberg quantum magnets without an external magnetic field can be generally described by the Boltzmann transport equation[6]

$$\frac{\partial f_k}{\partial t} + \mathbf{v}_k \cdot \nabla_{\mathbf{x}} f_k = -\frac{\partial f_k}{\partial t}\Big|_{\text{scattering}}, \tag{5}$$

which describes the dynamics of the out-of-equilibrium occupation function f_k at position $\mathbf{x}$ and time t, for all possible magnon states k ($k \equiv (\mathbf{q}, s)$, where $\mathbf{q}$ is the magnon wavevector and s is the magnon polarization). The second term on the left hand side of Eq. (5) describes advection processes of magnons, where $\mathbf{v_k}$ is the group velocity derived from the slope of the magnon dispersion. The right hand side term describes variations due to magnon scatterings. Here we only present a few key equations that help us to understand the underlying physics. Detailed derivation can be found in Ref. [7].

A. Two-in two-out magnon-magnon scattering

For two-dimensional Heisenberg antiferromagnets such as Rb_2MnF_4 , the spin-wave dispersion and damping are derived from the second order and higher order expansions of a spin operator Hamiltonian, respectively. At low temperatures, the spin order in a Heisenberg model is collinear. Magnetic anisotropy and the symmetry of spin operators restricts the leading-order magnon-magnon interactions to elastic pairwise collisions of magnons, *i.e.* $k + p \rightarrow s + r$. Three-magnon scatterings only become important when the non-collinear terms are non-negligible at elevated temperatures, *i.e.* close to the Néel temperature[8, 9], or for frustration. Derived from quantum perturbation theory, the scattering term in Eq. (5) for two-in/two-out processes is given as:

$$\begin{aligned}\frac{\partial f_k}{\partial t}\Big|_{scattering} &= \sum_{p,s} \frac{\pi H_E^2}{16 S^2 N^2} M_{22}(k,p,r,s)\delta(\omega_k + \omega_p - \omega_r - \omega_s)\Delta(\boldsymbol{q}_k + \boldsymbol{q}_p - \boldsymbol{q}_r - \boldsymbol{q}_s) \\ &\times \Big[(f_k+1)(f_p+1)f_r f_s - f_k f_p (f_r+1)(f_s+1)\Big],\end{aligned} \tag{6}$$

where $M_{22}(k, p, r, s)$ in Eq. (6) is referred to as the "matrix element" by Harris *et. al.*[9] and its derivation can be found in Ref. [10]. $H_E = 2JzS$, where J is the spin exchange strength, S is the

spin moment, and z is a quantum renormalization factor. The Kronecker delta Δ is zero unless its argument is zero or a reciprocal lattice vector, in which case it takes the value 1.

Microscopically, this two-in/two-out magnon-magnon interaction conserves crystal momentum, energy, and particle number [9] and macroscopically total energy and particle number are conserved. Non-conserving interactions (*i.e.*, changing particle number and/or energy within the magnon system) such as magnon-phonon scatterings are very weak in a gapped system at low temperatures, as the overlap of scattering phase space between phonon and magnon states is significantly reduced compared to gapless systems. Boundary scatterings are also very weak in a gapped system since the occupied states at low temperatures are primarily near the zone center and their group velocities are close to zero. Therefore, these non-conserving interactions occur at a time scale much slower than intrinsic magnon-magnon interactions. This prediction has been confirmed by high-precision neutron spin echo measurements of magnon linewidths of Rb_2MnF_4 and MnF_2, 2D and 3D Heisenberg antiferromagnets, respectively [8, 10].

B. Distribution function in a closed magnon system

We first restrict the solution of Eq. (5) to a closed system, where the interactions are limited to two-in two-out magnon-magnon scattering and no interactions with phonon bath or the environment through the boundaries. Starting with an initial distribution, f_k^i, deviating from the Bose-Einstein distribution, $f_k^{BE} = 1/(exp(\hbar\omega_k/k_BT_0) - 1)$, and following the derivation in Ref. [7] based on a eigendecomposition method, we obtained a closed form solution of the final distribution function given as

$$f_k(t \to \infty) = \frac{1}{\exp(\hat{e}_k) - 1} + \frac{\hat{e}_k}{4}\frac{1}{\sinh^2(\frac{\hat{e}_k}{2})}\Delta\hat{T} + \frac{1}{4}\frac{1}{\sinh^2(\frac{\hat{e}_k}{2})}\hat{\mu}, \tag{7}$$

where $\hat{e}_k = \hbar\omega_k/k_BT_0$, $\Delta\hat{T} = (T - T_0)/T_0$, and $\hat{\mu} = \mu/k_BT_0$. The values of T and μ are determined by the initial conditions through the conservation laws given as

$$\sum_k f_k(t \to \infty) = \sum_k f_k^i, \quad \sum_k \hbar\omega_k f_k(t \to \infty) = \sum_k \hbar\omega_k f_k^i. \tag{8}$$

It is easy to notice that Eq. (7) is merely the first order Taylor expansion of a displaced Bose-Einstein distribution function, $f^{displaced} = 1/(exp((\hbar\omega_k - \mu)/k_BT) - 1)$, around T_0 and $\mu_0 = 0$, where μ is often called chemical potential. This is not a surprising result. From a thermodynamic point of view, for a closed system of bosonic particles which conserves both particle number and total

energy, Eq. (7) maximizes the entropy given as

$$S = -k_B \sum_k [f_k \ln(f_k) - (1 + f_k)\ln(1 + f_k)]. \tag{9}$$

C. Nonequilibrium steady states under an external periodic drive

In a real dynamical system such as the driven-dissipative behaviors we observed in the INS measurements of Rb_2MnF_4, there always exists some slow damping mechanism. μ is time dependent and will eventually return to zero once the driving force disappears. To analyze the driven-dissipative behavior of the dynamics, we now add a phenomenological magnon-phonon coupling as a prototypical non-conserving interaction and external driving terms to Eq. (5), which then becomes

$$\frac{\partial f_k}{\partial t} + \mathbf{v}_k \cdot \nabla_{\mathbf{x}} f_k = -\frac{\partial f_k}{\partial t}\Big|_{\mathrm{m-m}} - \frac{f_k - f_k^{BE}(T)}{\tau_{ph-m}} + F_k(t), \tag{10}$$

where τ_{ph-m} is the relaxation time of magnons due to phonon-magnon interaction and $F_k(t)$ is an external driving source. Under the assumption of a closed boundary condition (no magnon flux in or out of the system), one is able to perform a volume integration to the BTE and use the divergence theorem to eliminate the spatial derivative term. From Eq. (10) and assuming $\tau_{ph-m} \gg \tau_{m-m}$, we follow the derivation in Ref. 17[7] and obtain a first-order ordinary differential equation for deviational magnon particle number, $\Delta N(t) = N(t) - N^{eq}(T_0)$, given as

$$\frac{d\Delta N}{dt} + \frac{\Delta N}{\tau_{ph-m}} = \Delta N^{Drive}(t), \tag{11}$$

where $\Delta N^{Drive}(t)$ is the external driving force.

Figure S10 shows the dynamics of $\Delta N(t)$ under a square-wave driving source, $\Delta N^{\mathrm{Drive}}(t) = \Delta N^{\mathrm{Drive}}(t+\mathcal{T})$, where $\mathcal{T}$ is the source period. Again we define P as the time between two excitation events. In Fig. S10, we fix the pulse width (PW) of the square-wave source. The ratio of τ_{ph-m} and PW determines the time-dependent behavior of $\Delta N(t)$. When $\tau_{ph-m} \ll$ PW (Fig. S10(a)), due to a fast magnon-phonon coupling time relative to the active pumping time, the magnon system quickly relaxes back to equilibrium once the driving source disappears. When $\tau_{ph-m} \sim$ PW (Fig. S10(b)), decay of the nonequilibrium magnon population is observed between two driving events and the decay time is determined by τ_{ph-m}. Observation of this dynamical behavior can be used to determine the coupling time of magnon-phonon interactions experimentally. When $\tau_{ph-m} \gg$ PW (Fig. S10(c)), the magnon system reaches a nonequilibrium steady state (NESS) and a nonequilibrium magnon

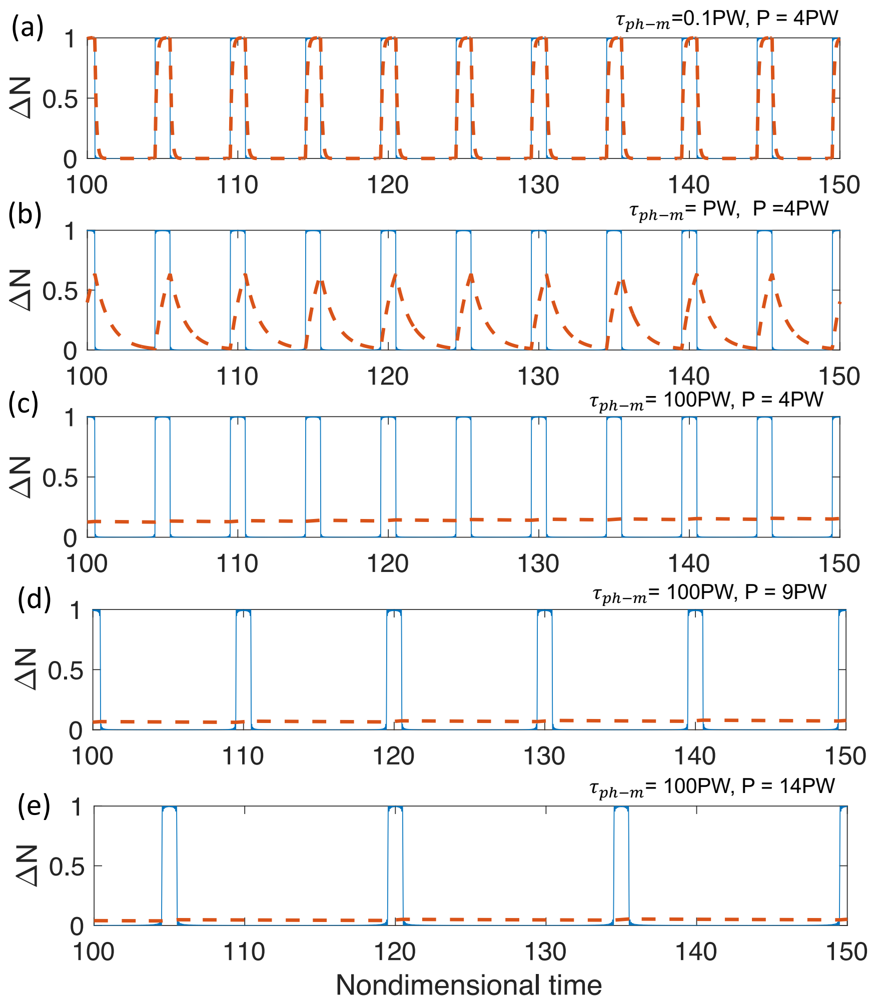

FIG. S10. Dynamical behavior of $\Delta N(t)$ (red dashed lines) with (a) $P/\tau_{ph-m} = 40$, (b) $P/\tau_{ph-m} = 4$, (c) $P/\tau_{ph-m} = 0.04$, (d) $P/\tau_{ph-m} = 0.09$, and (e) $P/\tau_{ph-m} = 0.14$ under a square-wave driving source (blue solid lines), $\Delta N^{\text{Drive}}(t) = \Delta N^{\text{Drive}}(t + \mathcal{T})$, where $\mathcal{T}$ is the source period. P is defined as the time between two excitation events, which is fixed here and is used to non-dimensionalize time. The ratio of τ_{ph-m} and PW determines the time-dependent behavior of $\Delta N(t)$.

population will be maintained as long as the driving source lasts. Once a nonequilibrium steady state condition is reached ($\tau_{ph-m} \gg$ PW), changing $\mathcal{T}$ or P only affects the nonequilibrium steady state value as shown in Figs. S10(c) - (e). Fig. S10(b) shows the nonequilibrium steady state value is inversely proportional to P. As $P \to \infty$, ΔN^{NESS} approaches zero and the system is at equilibrium.

From our INS measurements and the above theory, we can deduce that the non-equilibrium magnon population forms NESS's under a periodic external pumping. This means τ_{ph-m} is at least on the order of hundreds of milliseconds at 3.6 K. Although there is little information on

magnon-phonon scattering rates in quantum magnets in the literatures, the report of Bose-Einstein condensation of magnons in $CsMnF_3$[11], a Heisenberg antiferromagnet similar to Rb_2MnF_4, support such long lifetimes of non-equilibrium magnons.

III. QUANTUM THEORY ON BROKEN DETAILED BALANCE

So far, Boltzmann transport theory manages to explain why non-equilibrium steady states can be observed in Rb_2MnF_4 under the experimental conditions. However, as a classical theory, it will not be able to explain the broken detailed balance, which is a quantum mechanical property by nature. To understand why the detailed balance is broken as observed in the experiment, a non-equilibrium quantum transport theory has to be deployed.

To formulate the out-of-equilibrium processes illustrated in Fig. 3(c) of the main text, the pair correlation function, $\langle \hat{S}_i^\alpha(0)\hat{S}_{i'}^\beta(t)\rangle$, is first approximated under a linear spin wave theory in terms of two pairs of creation and annihiliation operators for a Heisenberg antiferromagnet, $\alpha^\dagger$, α, $\beta^\dagger$, and β. For example, the transverse component is written as

$$\langle \hat{S}_i^x(0)\hat{S}_{i'}^x(t)\rangle = A_{ii'}\langle\alpha^\dagger(0)\alpha(t)\rangle + B_{ii'}\langle\beta^\dagger(0)\beta(t)\rangle + B_{ii'}\langle\alpha(0)\alpha^\dagger(t)\rangle + A_{ii'}\langle\beta(0)\beta^\dagger(t)\rangle, \tag{12}$$

where $A_{ii'}$ and $B_{ii'}$ are coefficients determined by the constants in the spin Hamiltonian. In thermal equilibrium, the time-varying form of the operators $\alpha_l(t)$ and $\alpha_l^\dagger(t)$ are given as

$$\alpha_l(t) = N^{-1/2}\sum_{\mathbf{q}} \exp\{i(\mathbf{q}\cdot\mathbf{l} - \omega_{\mathbf{q}}t)\}a_{\mathbf{q}}, \tag{13}$$

$$\alpha_l^\dagger(t) = N^{-1/2}\sum_{\mathbf{q}} \exp\{-i(\mathbf{q}\cdot\mathbf{l} - \omega_{\mathbf{q}}t)\}a_{\mathbf{q}}^\dagger, \tag{14}$$

where $\langle\alpha_{\mathbf{q}}^\dagger\alpha_{\mathbf{q}}\rangle = n_{\mathbf{q}}^{BE}(T)$ gives the annihilation intensity and $\langle\alpha_{\mathbf{q}}\alpha_{\mathbf{q}}^\dagger\rangle = n_{\mathbf{q}}^{BE}(T) + 1$ gives the creation intensity.

When the underlying Hamiltonian is time-dependent and dynamical system is a driven-dissipative open system such as the non-equilibrium steady states as we observed in Rb_2MnF_4, the time dependence of $\alpha_l(t)$ and $\alpha_l^\dagger(t)$ is no longer given by Eqs. (13) & (14). We will need to solve quantum Langevin equation (QLE), which provides a direct description of the temporal behaviors of physical observables under the influence of deterministic and fluctuating forces. However, it will be extremely challenging to fully model the quantum transport of mixed states in many-body quantum system and is out of scope of the current work. Instead, here we will demonstrate how

detailed balance can be broken in a toy model where a single-mode harmonic oscillator is coupled to another single-mode harmonic oscillator with active pumping of the derived eigenmode of the coupled system. The former harmonic oscillator can be regarded as a spin mode and the latter harmonic oscillator is associated with atomic cloud, acting as a thermal bath. Here the derivation is adapted from the theory of non-equilibrium quantum phase transition in a driven optical cavity system. We will start with Hamiltonian of the toy model:

$$\mathcal{H} = \mathcal{H}_0 + \hbar\omega_b b^\dagger b + \hbar\omega_s \delta a^\dagger \delta a + \hbar\lambda(\delta a^\dagger + \delta a)(b^\dagger + b) \tag{15}$$

where $\mathcal{H}_0 = \hbar\omega_s a_0^\dagger a_0$ is the time-independent Hamiltonian and $a_0^\dagger$ and $\langle a_0^\dagger a_0\rangle = n^{BE}(T)$. δa is the first order expansion of a around a_0 such that

$$\langle a^\dagger(0)a(t)\rangle \approx \langle a_0^\dagger(0)a_0(t)\rangle + \langle \delta a^\dagger(0)\delta a(t)\rangle, \tag{16}$$

and the structure factor can be written as

$$\begin{aligned} S(\omega) = S_0(\omega) + \delta S(\omega) \;&\propto\; \langle a_0^\dagger a_0\rangle \delta(\omega + \omega_s) + \langle a_0 a_0^\dagger\rangle \delta(\omega - \omega_s) \\ &+\; \frac{1}{2\pi}\int_{-\infty}^{\infty} (\langle \delta a(-\omega')\delta a^\dagger(-\omega)\rangle + \langle \delta a^\dagger(\omega')\delta a(\omega)\rangle) d\omega'. \end{aligned} \tag{17}$$

Here, λ is the coupling strength between the bath and spin state. We can now write the deviational non-equilibrium Hamiltonian for the system as

$$\frac{\delta H}{\hbar} = \omega_b b^\dagger b + \omega_s \delta a^\dagger \delta a + \lambda(\delta a^\dagger + \delta a)(b^\dagger + b). \tag{18}$$

The corresponding quantum Langevin equation is given as

$$\frac{d}{dt}\begin{bmatrix} b \\ b^\dagger \\ \delta a \\ \delta a^\dagger \end{bmatrix} = M_0 \begin{bmatrix} b \\ b^\dagger \\ \delta a \\ \delta a^\dagger \end{bmatrix} \tag{19}$$

with the matrix

$$M_0 = \begin{bmatrix} -i\omega_b & 0 & -i\lambda & -i\lambda \\ 0 & i\omega_b & i\lambda & i\lambda \\ -i\lambda & -i\lambda & -i\omega_s & 0 \\ i\lambda & i\lambda & 0 & i\omega_s \end{bmatrix}. \tag{20}$$

Under the assumption of $\omega_b > \omega_s \gg \lambda$, diagonalization of M_0 via the transformation

$$S^{-1}M_0S = D = \begin{bmatrix} -i\omega_b & 0 & 0 & 0 \\ 0 & i\omega_b & 0 & 0 \\ 0 & 0 & -i\tilde{\omega}_s & 0 \\ 0 & 0 & 0 & i\tilde{\omega}_s \end{bmatrix} \tag{21}$$

with, neglecting terms of order, $\tilde{\omega}_s/\omega_s$,

$$S = \begin{bmatrix} 1 & 0 & \frac{-\lambda}{\omega_b}\sqrt{\frac{\omega_s}{\tilde{\omega}_s}} & \frac{-\lambda}{\omega_b}\sqrt{\frac{\omega_s}{\tilde{\omega}_s}} \\ 0 & 1 & \frac{-\lambda}{\omega_b}\sqrt{\frac{\omega_s}{\tilde{\omega}_s}} & \frac{-\lambda}{\omega_b}\sqrt{\frac{\omega_s}{\tilde{\omega}_s}} \\ \frac{-\lambda}{\omega_b} & \frac{\lambda}{\omega_b} & \frac{1+\tilde{\omega}_s/\omega_s}{2}\sqrt{\frac{\omega_s}{\tilde{\omega}_s}} & \frac{1-\tilde{\omega}_s/\omega_s}{2}\sqrt{\frac{\omega_s}{\tilde{\omega}_s}} \\ \frac{\lambda}{\omega_b} & \frac{\lambda}{-\omega_b} & \frac{1-\tilde{\omega}_s/\omega_s}{2}\sqrt{\frac{\omega_s}{\tilde{\omega}_s}} & \frac{1+\tilde{\omega}_s/\omega_s}{2}\sqrt{\frac{\omega_s}{\tilde{\omega}_s}} \end{bmatrix} \tag{22}$$

and

$$S^{-1} = \begin{bmatrix} 1 & 0 & \frac{\lambda}{\omega_b} & \frac{\lambda}{\omega_b} \\ 0 & 1 & \frac{\lambda}{\omega_b} & \frac{\lambda}{\omega_b} \\ \frac{-\lambda}{\omega_b}\sqrt{\frac{\omega_s}{\tilde{\omega}_s}} & \frac{\lambda}{\omega_b}\sqrt{\frac{\omega_s}{\tilde{\omega}_s}} & \frac{1+\omega_s/\tilde{\omega}_s}{2}\sqrt{\frac{\tilde{\omega}_s}{\omega_s}} & \frac{1-\omega_s/\tilde{\omega}_s}{2}\sqrt{\frac{\tilde{\omega}_s}{\omega_s}} \\ \frac{\lambda}{\omega_b}\sqrt{\frac{\omega_s}{\tilde{\omega}_s}} & \frac{-\lambda}{\omega_b}\sqrt{\frac{\omega_s}{\tilde{\omega}_s}} & \frac{1-\omega_s/\tilde{\omega}_s}{2}\sqrt{\frac{\tilde{\omega}_s}{\omega_s}} & \frac{1+\omega_s/\tilde{\omega}_s}{2}\sqrt{\frac{\tilde{\omega}_s}{\omega_s}} \end{bmatrix} \tag{23}$$

allows us to define the eigenmode operators d and c. Here $\tilde{\omega}_s = \omega_s\sqrt{1 - 4\lambda^2/(\omega_b\omega_s)}$ is the eigenfrequency of the quasi-particle we are interested in.

The assumption $\omega_s/\omega_b < 1$ is introduced purely for analytical convenience, indicating that the energy of the bath mode exceeds that of the spin mode. This hierarchy allows for a controlled expansion of the transformation matrix S, enabling us to neglect higher-order terms in $\tilde{\omega}_s/\omega_s$ and to obtain closed-form analytical expressions for the eigenmodes. Importantly, this assumption is not intended to impose a physical constraint. In realistic systems, multiple bath and spin modes are present, and no simple one-to-one energy relation applies. Diagonalization of the coupled Hamiltonian yields the eigenmodes of the interacting system. Independent of the specific assumption, $\omega_s/\omega_b < 1$, the normal modes, c and $c^\dagger$, are expressed as a linear combination of b, $b^\dagger$, δa, and $\delta a^\dagger$. While the assumption modifies the numerical values of the coefficients in these linear combinations, it does not alter the formal structure of the resulting equations.

Note that S^{-1} is normalized such that the bosonic commutation relations for $d^\dagger$ and $c^\dagger$ defined as

$$\begin{bmatrix} d \\ d^\dagger \\ c \\ c^\dagger \end{bmatrix} = S^{-1} \begin{bmatrix} b \\ b^\dagger \\ \delta a \\ \delta a^\dagger \end{bmatrix} \tag{24}$$

are valid. The transformation present above is correct up to terms of order ω_s/ω_b. For $\lambda \to 0$, Eq. (24) yields $c \to \delta a$ and $d \to b$. We now have the decoupled Langevin equations given as

$$\frac{d}{dt}\begin{bmatrix} d \\ d^\dagger \\ c \\ c^\dagger \end{bmatrix} = \frac{d}{dt} S^{-1} \begin{bmatrix} b \\ b^\dagger \\ \delta a \\ \delta a^\dagger \end{bmatrix} = S^{-1} M_0 S S^{-1} \begin{bmatrix} b \\ b^\dagger \\ \delta a \\ \delta a^\dagger \end{bmatrix}. \tag{25}$$

We now formally introduce a damping rate γ of the c-mode. Here we assume that $\gamma \ll \lambda$. This assumption allows us to first couple the spin mode, δa, to the atomic mode, b. This results in a new eigenmode of the spin system which describes the quasi-particles of the slow interacting system. Only thereafter we introduce the damping of the eigenmode at rate γ. We now define input operators with correlation functions $\langle c_{in}^\dagger(t) c_{in}(t')\rangle = 0$ and $\langle c_{in}(t) c_{in}^\dagger(t')\rangle = \delta(t - t')$. Accordingly, Eq. (26) becomes

$$\dot{c} = (-i\tilde{\omega}_s - \gamma)c + \sqrt{2\gamma} c_{in}. \tag{26}$$

The solution to Eq. (26) is given by

$$c(t) = \int_0^t e^{(-i\tilde{\omega}_s - \gamma)(t-t')} \sqrt{2\gamma} c_{in}(t') dt'. \tag{27}$$

Applying a backtransformation using the matrix S, and move to the frequency space:

$$\delta a(\omega) = \frac{\lambda}{\omega_b}\sqrt{\frac{\omega_s}{\tilde{\omega}_s}}(-d(\omega) + d^\dagger(-\omega)) + \frac{1}{2}\sqrt{\frac{\tilde{\omega}_s}{\omega_s}}\left[\left(1 + \frac{\omega_s}{\tilde{\omega}_s}\right)c(\omega) + \left(1 - \frac{\omega_s}{\tilde{\omega}_s}\right)c^\dagger(-\omega)\right] \tag{28}$$

$$\delta a^\dagger(\omega) = \frac{\lambda}{\omega_b}\sqrt{\frac{\omega_s}{\tilde{\omega}_s}}(d(-\omega) - d^\dagger(\omega)) + \frac{1}{2}\sqrt{\frac{\tilde{\omega}_s}{\omega_s}}\left[\left(1 - \frac{\omega_s}{\tilde{\omega}_s}\right)c(-\omega) + \left(1 + \frac{\omega_s}{\tilde{\omega}_s}\right)c^\dagger(\omega)\right]. \tag{29}$$

Using the Langevin equation in Fourier space, the expectation value of deviational particle number is written as

$$\begin{aligned} \langle \delta a^\dagger(\omega')\delta a(\omega)\rangle &= \frac{\lambda^2}{\omega_b^2}\frac{\omega_s}{\tilde{\omega}_s}(\langle d^\dagger(\omega')d(\omega)\rangle + \langle d(-\omega')d^\dagger(-\omega)\rangle) \\ &+ \frac{\tilde{\omega}_s}{4\omega_s}\left[\left(1 + \frac{\omega_s}{\tilde{\omega}_s}\right)^2 \langle c^\dagger(\omega)c(\omega)\rangle + \left(1 - \frac{\omega_s}{\tilde{\omega}_s}\right)^2 \langle c(-\omega)c^\dagger(-\omega)\rangle\right] \\ &\approx \frac{\tilde{\omega}_s}{2\omega_s}\left(1 - \frac{\omega_s}{\tilde{\omega}_s}\right)^2 \frac{\gamma\delta(\omega - \omega')}{(i\omega' + i\tilde{\omega}_s + \gamma)(-i\omega - i\tilde{\omega}_s + \gamma)}. \end{aligned} \tag{30}$$

Similarly, we have

$$\langle\delta a(\omega')\delta a^{\dagger}(\omega)\rangle \approx \frac{\tilde{\omega}_s}{2\omega_s}\left(1+\frac{\omega_s}{\tilde{\omega}_s}\right)^2 \frac{\gamma\delta(\omega-\omega')}{(-i\omega'+i\tilde{\omega}_s+\gamma)(i\omega-i\tilde{\omega}_s+\gamma)}. \tag{31}$$

In Eqs. (30) & (31), we set $\langle d^{\dagger}(\omega')d(\omega)\rangle$ and $\langle d(\omega')d^{\dagger}(\omega)\rangle$ to zero as fluctuation in the atomic modes would be vanishingly small at low temperatures.

We can now explicitly calculate the deviational part of the structure factor given as

$$\delta S(\omega) \propto \frac{1}{2\pi}\int_{-\infty}^{\infty}\langle\delta a(-\omega')\delta a^{\dagger}(-\omega)\rangle + \langle\delta a^{\dagger}(\omega')\delta a(\omega)\rangle d\omega' = \frac{1}{2\pi}\left(\frac{\tilde{\omega}_s}{\omega_s}+\frac{\omega_s}{\tilde{\omega}_s}\right)\frac{\gamma}{(\omega+\tilde{\omega}_s)^2+\gamma^2} \tag{32}$$

We notice that $\delta S(\omega)$ appears as a Lorentzian peak that is centered around $\omega = -\tilde{\omega}_s$. In a periodically driven dissipative open quantum system, the active pumping of quasiparticles gives rise to the extra intensity on the annihilation side of the structure factor.

---

[1] G. L. Squires, *Introduction to the theory of thermal neutron scattering* (Dover Publications, Mineola, N.Y, 1996).

[2] A. Furrer, J. Mesot, and T. Strassle, *Neutron Scattering in Condensed Matter Physics*, Vol. 04 (WORLD SCIENTIFIC, 2009).

[3] T. Huberman, R. Coldea, R. A. Cowley, D. A. Tennant, R. L. Leheny, R. J. Christianson, and C. D. Frost, Two-magnon excitations observed by neutron scattering in the two-dimensional spin- 5 2 heisenberg antiferromagnet rb 2 mn f 4, **72**, 014413 (2005).

[4] J. Y. Y. Lin, F. Islam, G. Sala, I. Lumsden, H. Smith, M. Doucet, M. B. Stone, D. L. Abernathy, G. Ehlers, J. F. Ankner, and G. E. Granroth, Recent developments of MCViNE and its applications at SNS, Journal of Physics Communications **3**, 085005 (2019).

[5] C. Hua, D. A. Tennant, A. T. Savici, V. Sedov, G. Sala, and B. Winn, Implementation of a laser–neutron pump–probe capability for inelastic neutron scattering, Review of Scientific Instruments **95**, 033902 (2024).

[6] T. Liu, W. Wang, and J. Zhang, Collective induced antidiffusion effect and general magnon boltzmann transport theory, Physical Review B **99**, 214407 (2019).

[7] C. Hua, L. Lindsay, Y. Shinohara, and D. A. Tennant, Dynamics of nonequilibrium magnons in gapped Heisenberg antiferromagnets, Physical Review B **109**, 054306 (2024).

[8] A study of the quantum classical crossover in the spin dynamics of the 2D S=5/2 antiferromagnet Rb2MnF4: neutron scattering, computer simulations, and analytic theories, **2008**.

[9] A. B. Harris, D. Kumar, B. I. Halperin, and P. C. Hohenberg, Dynamics of an antiferromagnet at low temperatures: Spin-wave damping and hydrodynamics, Phys. Rev. B **3**, 961 (1971).

[10] S. P. Bayrakci, D. A. Tennant, P. Leininger, T. Keller, M. C. R. Gibson, S. D. Wilson, R. J. Birgeneau, and B. Keimer, Lifetimes of Antiferromagnetic Magnons in Two and Three Dimensions: Experiment, Theory, and Numerics, Physical Review Letters **111**, 10.1103/PhysRevLett.111.017204 (2013).

[11] Y. M. Bunkov, E. M. Alakshin, R. R. Gazizulin, A. V. Klochkov, V. V. Kuzmin, T. R. Safin, and M. S. Tagirov, Discovery of the classical Bose-Einstein condensation of magnons in solid antiferromagnets, JETP Letters **94**, 68 (2011).
